# Predictors of evidence-based psychotherapy initiation among veterans with co-occurring PTSD and substance use disorder

**DOI:** 10.3389/fpsyt.2024.1432361

**Published:** 2024-08-16

**Authors:** Vanessa C. Somohano, David Cameron, Meaghan M. Lewis, Allison O’Neill, Rachel Phillips, Joshua Kaplan, Maya E. O’Neil

**Affiliations:** ^1^ Center to Improve Veteran Involvement in Care, VA Portland Healthcare System, Portland, OR, United States; ^2^ Department of Psychiatry, Oregon Health & Science University, Portland, OR, United States; ^3^ Integrative Neurology and Intraoperative Neuromonitoring, Oregon Health & Science University, Portland, OR, United States; ^4^ Department of Medical Informatics and Clinical Epidemiology, Oregon Health & Science University, Portland, OR, United States

**Keywords:** health service utilization, PTSD, substance use disorder, dual-diagnosis, veterans, evidence-based psychotherapy

## Abstract

**Objective:**

To compare initiation of PTSD evidence-based psychotherapy (EBP) between Veterans with and without a co-occurring substance use disorder (SUD), and identify factors associated with EBP initiation among Veterans with PTSD-SUD.

**Method:**

A national sample of Veterans with PTSD (*n* = 301,872) and PTSD-SUD (*n* = 94,515) were identified from VA Electronic Health Record data. Treatment initiation was defined as having at least one mental health encounter associated with Cognitive Processing Therapy or Prolonged Exposure therapy. Generalized estimating equations were used to compare EBP initiation between Veterans with and without co-occurring SUD, and to identify patient- and facility characteristics associated with EBP initiation among Veterans with PTSD-SUD.

**Results:**

The majority of Veterans were 30 – 44 years old, male sex, and Non-Hispanic White. No significant differences were observed in EBP initiation between Veterans with and without a co-occurring SUD (*OR*=1.00, *p*=0.985). Among Veterans with PTSD-SUD, co-occurring bipolar disorder (*OR*=0.83, *p*=.000), co-occurring psychotic disorder (*OR*=0.69, *p*=.000), service connection (*OR*=0.94, *p*=.001), female sex (*OR*=0.87, *p*=.000), and being 60 years or older (*OR*=0.57, *p*=.000) were associated with a reduced likelihood of initiating a PTSD EBP. Having a co-occurring anxiety disorder (*OR*=1.06, *p*=.020), MST history (*OR*=1.95, *p*=.000), and high risk for suicide (*OR*=1.15, *p*=.000) were associated with an increased likelihood of initiating EBP.

**Discussion:**

These findings support VA provision of EBP for Veterans with PTSD regardless of the presence of co-occurring SUD. Identifying characteristics that increase or reduce the likelihood of EBP initiation may provide insight into treatment pathways and subgroups warranting augmented outreach.

## Introduction

Substance use disorder (SUD) commonly co-occurs with Posttraumatic Stress Disorder (PTSD), and both disorders are particularly prevalent among Veterans (1). Approximately one in five Veterans with PTSD have a co-occurring SUD, and 63% of Veterans with SUD have co-occurring PTSD (1). Compared to Veterans with either disorder alone, dually diagnosed Veterans demonstrate poorer treatment outcomes, increased rates of treatment dropout, higher rates of health stressors, more severe medical and psychiatric symptoms, increased risk of legal problems, and higher rates of houselessness (2–4). PTSD and SUD symptoms observed in Veterans who participated in 20^th^ century conflicts led to an increase in PTSD research beginning in the 1990s (5, 6), and has since enhanced the Veterans Healthcare Administration’s (VHAs) ability to identify and effectively respond to PTSD and co-occurring SUD.

Through robust research efforts, evidence-based psychotherapy (EBP) has emerged for the treatment of PTSD. EBP considered “first-line” treatments for PTSD include Cognitive Processing Therapy (CPT) (7) and Prolonged Exposure (PE) (8); both have strong evidence of efficacy (9) and are broadly disseminated across the Veterans Healthcare Administration (VHA). Until recently, clinical practice guidelines recommended treating SUD before PTSD for individuals with both disorders (10). This was driven by the symptom exacerbation hypothesis, which posits that PTSD symptoms will exacerbate SUD symptoms if treated concurrently (11, 12). However, recent research has demonstrated that EBP for PTSD is safe and equally effective for those with co-occurring SUD (13), and do not exacerbate PTSD symptoms among those with co-occurring SUD more than those with PTSD alone (14, 15). Additionally, substance craving and distress associated with exposure-based PTSD interventions are not predictive of future elevated PTSD and SUD symptoms (13). Moreover, evidence suggests that habituation to distress and cravings between EBP sessions predicts reductions in PTSD and substance use outcomes (16), indicating that skills learned during EBP can reduce both PTSD and SUD symptoms together if enacted between sessions. Overall, research suggests individuals with PTSD-SUD are as likely to benefit from EBP for PTSD as those with PTSD alone, and are not at greater risk for symptom exacerbation when engaging in EBP (15).

Despite evidence supporting the safety and efficacy of EBP for PTSD for dually diagnosed individuals, little is known about EBP utilization in this population. This may be because clinicians are hesitant to administer EBP with dually diagnosed individuals due to misperceptions of appropriateness of trauma-focused therapies and lack of training in EBP for PTSD (17, 18). Some clinical trials have also historically excluded individuals with co-occurring SUD due to higher dropout rates in research participation or to have a more selective inclusion criteria (i.e., PTSD only) (10). This has limited the number of high quality studies supporting the use of EBP for dually diagnosed individuals. In addition to often excluding those with co-occurring SUD from clinical trials, rates of EBP initiation in dually diagnosed individuals has not been well studied. Only one retrospective study using a national sample of justice-involved Veterans with PTSD (*N* = 27,857) found that having a co-occurring SUD facilitated initiation of PTSD treatment (4). However, no largescale studies using samples representative of the general VA-using patient population have explored EBP utilization among those with PTSD-SUD compared to those with PTSD only. Subsequently, predictors of EBP utilization are largely unknown among Veterans with PTSD-SUD, although patient and health service predictors of EBP utilization among those with *PTSD alone* has been established (19–21).

Those with PTSD-SUD are at increased risk of negative psychiatric and functional outcomes, and greater clinical and functional impairment (2–4, 22). Thus, distinguishing which factors increase and reduce the likelihood of EBP initiation among those with PTSD-SUD may improve access to high quality treatments for this population by informing strategies that work better for subgroups with PTSD-SUD, thereby enhancing EBP engagement. The aims of this study were twofold:

1) To compare rates of EBP initiation between Veterans with PTSD-SUD versus PTSD only.

2) To identify patient and health service factors associated with EBP initiation among Veterans with PTSD-SUD.

Given literature showing that clinicians often view EBP as inappropriate for those with co-occurring SUD (17), we hypothesized that Veterans with PTSD only would initiate EBP at a higher rate than those with PTSD-SUD. Further, based on predictors of EBP initiation among Veterans with PTSD only (19–21), we hypothesized that Veterans of a younger age, having a co-occurring serious mental illness (SMI) such as bipolar or psychotic spectrum disorders, being of a minoritized race and ethnicity, and receiving services in a rural location would be associated with lower EBP initiation among those with PTSD-SUD. We also hypothesized that having a service connected disability, a history of military sexual trauma (MST), being at high risk for suicide, and receiving services at a high complexity level site would be associated with greater initiation of EBP among those with PTSD-SUD.

## Method

### Participants and procedure

Retrospective demographic, diagnosis, encounter, and EBP template information was obtained for each participant from the Corporate Data Warehouse (CDW), a national database of VHA Electronic Health Record (EHR) data. All data collected was part of routine clinical care procedures; thus, the VA Portland Healthcare System IRB approved a waiver for informed consent.

A cohort of Veterans with PTSD only (*n* = 301,872) or PTSD-SUD diagnosis (*n* = 94,515) between January 1st, 2017 and December 31st, 2019 were identified. A PTSD diagnosis was defined as having at least two outpatient International Classification of Diseases (ICD)-9 or ICD-10 codes associated with a mental health encounter at a VA clinic within a 90-day period. A SUD diagnosis was defined having at least one inpatient ICD-9 or ICD-10 code, or at least two outpatient ICD-9 or ICD-10 codes, associated with a mental health encounter for SUD 12 months prior to the first mental health encounter with a PTSD ICD-9 or ICD-10 code. An encounter is defined as a documented visit in the EHR that occurred between a patient and a healthcare provider.

As part of a national initiative to disseminate EBP for PTSD over the last decade (23), the VHA introduced EHR templates to document the use of CPT or PE therapy sessions, which are protocoled and standardized evidence-based psychotherapies (EBPs) for PTSD. The use of EBP templates became mandatory in the 2015 fiscal year (24). EBP templates generate “health factors”, or trackable codes within the EHR describing which treatment components occurred in session, session number, and type of therapy administered; information captured by health factors are stored in the CDW. Although nation-wide rollout of EBP templates occurred in 2014, robust use of templates became more commonplace in 2017 (25), which provided more reliable data; thus, we limited our inclusion criteria to encounters that occurred after January 1^st^, 2017. Further, encounters that occurred after December 31, 2019 were excluded to control for possible confounding effects of SARS-CoV-2 (COVID-19) healthcare and policy changes. Encounters associated with group CPT and Eye Movement Desensitization and Reprocessing (EMDR) therapy were not included because there are currently no standardized EHR templates for the intervention. Participants were also excluded if they died before the end of 2019.

Treatment initiation was defined as having at least one mental health encounter associated with individual CPT or individual PE; participants were categorized as initiating *either* CPT *or* PE. If participants initiated both EBPs during their episode of care, the first EBP in which the participant initiated was used for classification. Patients were excluded if they met diagnostic criteria prior to 2017, ensuring that patients receiving EBP were treatment naïve.

### Measures

#### Patient-level factors

Patient-level factors included in logistic regression models were sex (male, female), age (18-29, 30-44, 45-59, 60+), race (White, Black, Asian, Native American/Alaska Native, Pacific Islander, race unknown), ethnicity (Hispanic/non-Hispanic), co-occurring mood disorders (unipolar depression, other anxiety disorders), co-occurring SMI (bipolar spectrum disorders, psychotic disorders), service connection status, history of MST and high suicide risk. Of note, race is a political and social construct that serves as a proxy for the impact of racist practices and structural inequality, and is not a biological variable; thus, race is examined in the current study with this premise in mind. Additionally, individuals identifying their gender or sex as anything other than “male” or “female” was not tracked in the EHR system at the time of data collection, and thus, more nuanced gender identity data are not accessible in our data. A service connected disability refers to a formal VA disability status in which a Veteran’s VA-rated physical or mental health conditions were caused by or during their military service. High suicide risk is defined as any patient who had a suicide safety plan health factor or note title, or who had a high risk for suicide flag in the EHR in the 3 years prior to their PTSD diagnosis.

#### Facility-level factors

Locality (i.e., rural or urban) and hospital complexity were facility-level factors included in the logistic regression models. Locality was identified by the area code in which a VA facility was located (26). Facility complexity is determined by The Clinical Complexity Index, which designates VHA facilities into five classification levels: 1a, 1b, 1c, 2, 3. A classification of 1a denotes the most complex facilities, and complexity level 3 facilities are the least complex (27). Highest complexity facilities have the capacity to serve a greater volume of patients, the highest risk patients and specialty care needs, and have infrastructure supporting large research and teaching programs (27).

### Data analysis

Descriptive statistics are presented in **Table 1**. We calculated the proportion of Veterans with PTSD-SUD and PTSD only who initiated EBP. For aim 1, we used general estimating equations (GEE) with a logit link to compare the proportion of Veterans with PTSD only versus Veterans with co-occurring PTSD-SUD who initiated EBP. For aim 2, we used GEE with a logit link to identify patient- and facility-level factors associated with the likelihood of EBP initiation among those with PTSD-SUD. Missing data were handled by listwise deletion. GEE models were specified with a compound symmetry correlation structure to account for correlations between patients within VHA facilities. All analyses were performed in SAS, version 9.4 (SAS Institute, Cary, NC).

**Table 1:** Sample characteristics among Veterans, stratified by SUD co-morbidity.

Sample Characteristic	Overall *N* = 396,387		PTSD-only *n* = 301,872		PTSD-SUD *n* = 94,515	
	N	%	n	%	n	%
Age category						
18 – 29	46,436	12	34,551	11	11,885	13
30 – 44	141,072	36	105,254	35	35,818	38
45 – 59	102,597	26	76,089	25	26,508	28
60+	106,282	27	85,978	28	20,304	21
Sex (Female)	63,705	16	53,979	18	9,726	10
Race						
Non-Hispanic White	254,746	64	195,174	65	59,572	63
Black/African American	97,085	24	71,882	24	25,203	27
American Indian/Alaska Native	4,652	1.2	3,375	1.5	1,277	1.4
Asian	5,549	1.4	4,670	1.5	879	0.9
Pacific Islander	4,600	1.2	3,651	1.2	949	1.0
Multiracial	5,348	1.3	4,111	1.4	1,237	1.3
Missing	24,407	6.2	19,009	6.3	5,398	5.7
Ethnicity (Latinx/Hispanic)	40,469	10	31,317	10	9,152	10
Co-morbid mood disorder						
Depressive disorders	196,238	50	139,885	46	56,353	60
Other anxiety disorders	158,277	40	112,166	37	46,111	49
Co-morbid SMI						
Bipolar disorders	35,213	8.9	20,167	6.7	15,046	16
Psychotic disorders	10,211	2.6	5,231	1.7	4,980	5.3
Service connected	269,020	68	213,421	71	55,599	59
MST	56,904	14	43,842	15	13,062	14
High suicide risk	43,562	11	21,103	7.0	22,459	24
Locality (Rural)	22,304	5.6	16,805	5.6	5,499	5.8
Facility complexity						
1a	191,967	48	146,344	48	45,623	48
1b	74,302	19	57,034	19	17,286	18
1c	50,845	13	39,350	13	11,495	12
2	36,188	9.1	27,964	9.3	8,224	8.7
3	43,085	11	31,180	10	11,905	13
Initiated EBP	55,730	14	42,196	14	13,534	14

SUD, substance use disorder; SMI, serious mental illness; MST, military sexual trauma; EBP, evidence-based psychotherapy.

### Transparency and openness

This study was approved by the VA Portland Healthcare System IRB, study #4460. We have reported all data exclusions and all measures in the study, and followed JARS (28). Raw data were generated at the VHA CDW. Derived data and data code supporting the findings of this study are not publicly available because the VA requires an approved data access request to access VA data; however, data can be made available from the corresponding author on request. This study’s design and its analysis were not pre-registered.

## Results

There were *N* = 396,387 Veterans who received either a PTSD (*n* = 301,872) or a PTSD-SUD diagnosis (*n* = 94,515) between 2017 – 2019. Most Veterans were between the ages of 30 – 44 years old (*n* = 141,072; 36%), male sex assigned at birth (*n* = 332,682; 84%), and identified as Non-Hispanic White (*n* = 254,746; 64%). Half of the sample received a diagnosis for co-occurring depressive disorder (*n* = 196,238; 50%), and 40% (*n* =158,277) received a diagnosis for an anxiety disorder. There was a higher percentage of individuals diagnosed with bipolar disorder among those with PTSD-SUD (*n* = 15,046; 16%) compared to the those with PTSD only (*n* = 20,167; 6.7%), and 2.6% (*n* = 10,211) of the sample received a diagnosis for a psychotic disorder, with higher percentages among those with PTSD-SUD (*n* = 4,980; 5.3%) compared to those with PTSD only (*n* = 5,231; 1.7%).

Those with PTSD-SUD also had higher rates of being at a high risk for suicide (*n* = 22,459; 24%) compared to those with PTSD-only (*n* = 21,103; 7%). Those who screened positive for MST comprised 14% (*n* = 56,904) of the entire sample and was similar between those with PTSD and PTSD-SUD. Sixty-eight percent of the entire sample had a service connected disability, with a higher proportion of service connection among those with PTSD only (*n* = 213,421; 71%) compared to those with PTSD-SUD (*n* = 55,599; 59%). Most Veterans received services at high complexity hospital sites (*n* = 317,114; 80%) within an urban setting (*n* = 374,083; 94.4%). Only 14% (*n* = 55,730) initiated an EBP for PTSD, with 80% of those (*n* = 44,584) opting to initiate CPT (rather than PE). See **Table 1** for full sample characteristics.

Logistic regression analyses revealed no significant differences in the proportion of Veterans who initiated EBP treatment between those with PTSD only versus PTSD-SUD (*OR* = 1.00, 95%CI: 0.96, 1.04) (See **Table 2**). Using the subset of data with *only* Veterans with PTSD-SUD, a second logistic regression analysis revealed several patient and health service factors that increased or reduced the likelihood of treatment initiation. Having co-occurring bipolar disorder *(OR* = 0.83, 95%CI: 0.77, 0.89), or psychotic disorders (*OR* = 0.69, 95%CI: 0.58, 0.8); having a service connected disability (*OR* = 0.94, 95%CI: 0.9, 0.98); being of female sex (*OR* = 0.87, 95%CI: 0.79, 0.95); and being 60 years or older (*OR* = -0.57, 95%CI: 0.49, 0.65) were associated with a reduced likelihood of initiating PTSD EBP. Factors associated with an increased likelihood of initiating EBP included having an anxiety disorder (OR = 1.06, 95%CI: 1.01, 1.11); having MST history (OR = 1.95, 95%CI: 1.89, 2.01); and being at high risk for suicide (OR = 1.15, 95%CI: 1.09, 1.21). See **Table 3** for regression coefficients.

**Table 2:** Regression coefficients for EBP initiation between Veterans with PTSD-only versus PTSD-SUD.

Parameter	*B*	*SE*	*OR*	95% CI	*p*
Intercept	-1.615	0.073	0.20	(0.06, 0.34)	< 0.001
Age category (Reference: 18 – 29)					
30 - 44	0.036	0.014	1.04	(1.01, 1.07)	0.009
45 - 59	0.030	0.018	1.03	(0.99, 1.07)	0.099
60+	-0.533	0.027	0.59	(0.54, 0.64)	< 0.001
Sex (Reference: Male)	-0.003	0.018	1.00	(0.96, 1.04)	0.830
Race (Reference: Non-Hispanic White)					
AI/AN	0.078	0.041	1.08	(1, 1.16)	0.058
Asian	-0.038	0.039	0.96	(0.88, 1.04)	0.335
Black	0.025	0.021	1.03	(0.99, 1.07)	0.238
Missing	-0.020	0.020	0.98	(0.94, 1.02)	0.299
Multi-race	-0.046	0.034	0.95	(0.88, 1.02)	0.176
Pacific Islander	-0.088	0.049	0.92	(0.82, 1.02)	0.076
Ethnicity (Reference: Not Hispanic)	0.049	0.017	1.05	(1.02, 1.08)	0.003
Mood disorders (Reference: No mood disorder)					
Unipolar depression	-0.151	0.013	0.86	(0.83, 0.89)	< 0.001
Anxiety	-0.005	0.012	0.99	(0.96, 1.02)	0.697
SMI (Reference: No SMI)					
Bipolar disorders	-0.223	0.021	0.8	(0.76, 0.84)	< 0.001
Psychotic disorders	-0.442	0.041	0.64	(0.56, 0.72)	< 0.001
Service connected (Reference: Not service connected)	-0.041	0.011	0.96	(0.94, 0.98)	< 0.001
MST (Reference: No MST)	0.548	0.025	1.73	(1.68, 1.78)	< 0.001
High suicide risk (Reference: No high risk)	0.104	0.025	1.11	(1.06, 1.16)	< 0.001
Locality (Reference: Urban)	0.037	0.132	1.04	(0.78, 1.3)	0.779
Facility complexity (Reference: 1a)					
1b	0.132	0.142	1.14	(0.86, 1.42)	0.779
1c	-0.287	0.141	0.75	(0.47, 1.03)	0.354
2	0.112	0.147	1.12	(0.83, 1.41)	0.448
3	0.036	0.132	1.04	(0.78, 1.3)	0.781
Substance Use Disorder (Reference: No SUD)	-0.0003	0.019	1	(0.96, 1.04)	0.985

N = 396,387. Parameters adjusted for demographic variables. AI/AN, American Indian/Alaska Native; MST, military sexual trauma; SMI, serious mental illness; SUD, Substance Use Disorder.

**Table 3:** Regression coefficients for predictors of PTSD EBP initiation among Veterans with PTSD-SUD.

Parameter	*B*	*SE*	*OR*	95% CI	*p*
Intercept	-1.78	0.06	0.17	(0.05, 0.29)	0.000
Age category (Reference: 18 - 29)					
30 - 44	0.018	0.028	1.02	(0.96, 1.08)	0.515
45 - 59	-0.030	0.032	0.97	(0.91, 1.03)	0.355
60+	-0.557	0.040	0.57	(0.49, 0.65)	0.000
Sex (Reference: Male)	-0.144	0.040	0.87	(0.79, 0.95)	0.000
Race (Reference: White)					
AI/AN	0.074	0.076	1.08	(0.93, 1.23)	0.335
Asian	-0.112	0.079	0.89	(0.73, 1.05)	0.162
Black	-0.01423	0.031	0.99	(0.93, 1.05)	0.654
Missing	-0.026	0.041	0.97	(0.89, 1.05)	0.526
Multi-race	-0.057	0.064	0.94	(0.81, 1.07)	0.375
Pacific Islander	-0.141	0.077	0.87	(0.72, 1.02)	0.067
Ethnicity (Reference: Not Hispanic)	0.020	0.038	1.02	(0.95, 1.09)	0.598
Mood disorder (Reference: No mood disorder)					
Unipolar depression	-0.028	0.026	0.97	(0.92, 1.02)	0.285
Anxiety	0.056	0.024	1.06	(1.01, 1.11)	0.020
SMI (Reference: No SMI)					
Bipolar disorders	-0.183	0.032	0.83	(0.77, 0.89)	0.000
Psychotic disorders	-0.377	0.055	0.69	(0.58, 0.80)	0.000
Service connected (Reference: Not service connected)	-0.067	0.0194	0.94	(0.90, 0.98)	0.001
MST (Reference: No MST)	0.669	0.033	1.95	(1.89, 2.01)	0.000
High suicide risk (Reference: No suicide risk)	0.140	0.031	1.15	(1.09, 1.21)	0.000
Locality (Reference: Urban)	0.065	0.17	1.07	(0.73, 1.41)	0.711
Facility complexity (Reference: 1a)					
1b	0.227	0.125	1.25	(1.00, 1.50)	0.071
1c	-0.054	0.157	0.95	(0.64, 1.26)	0.734
2	0.133	0.145	1.14	(0.86, 1.42)	0.362
3	0.141	0.139	1.15	(0.88, 1.42)	0.312

N = 94,515. AI/AN, American Indian/Alaska Native; MST, military sexual trauma; SMI, serious mental illness.

## Discussion

The aims of the current study were to determine whether the likelihood of EBP initiation was similar for Veterans with PTSD-SUD compared to those with PTSD alone, and to identify patient- and health-service factors associated with PTSD EBP initiation among Veterans with PTSD-SUD. Contrary to our first hypothesis, Veterans with PTSD only were statistically just as likely to initiate EBP for PTSD as were those with PTSD-SUD. This indicates that Veterans with PTSD-SUD being treated within VA settings may be provided with similar opportunities for EBP utilization. The VHA’s recent investment in disseminating EBP for PTSD may buffer some of the previously cited barriers to EBP initiation for those with PTSD-SUD. For example, the VHA offers free EBP trainings to qualified providers, continuing education courses on recent research of EBP implementation to subpopulations of Veterans, including those with PTSD-SUD, and consultation services for clinical and professional support of providers who are implementing EBP. Moreover, VHA requires that EBP is used as first-line interventions for PTSD (23); therefore, clinicians may be more likely to utilize these treatments even if a patient has co-occurring SUD.

Based on research with PTSD only samples, we hypothesized that being younger, having a co-occurring SMI such as bipolar disorder or a psychotic disorder, and being of a minoritized race and ethnicity would be associated with lower EBP initiation. We also hypothesized that having a service connected disability, a history of MST, and indicators for being at high risk for suicide in the medical record would be associated with an increased probability of EBP initiation among those with PTSD-SUD. Our hypotheses regarding factors associated with lower odds of initiation were partially supported. As predicted, those with a co-occurring diagnosis of bipolar disorder or a psychotic disorder was associated with a reduced likelihood of initiating EBP. Unexpectedly, female sex assigned at birth and older age (above age 60) were also both associated with a reduced likelihood of EBP initiation. Contrary to our hypotheses, having a service connected disability was also associated with a reduced likelihood of EBP initiation. Our hypotheses regarding factors associated with increased odds of initiating EBP were also partially confirmed. A history of MST and being at an increased risk for suicide were associated with increased odds of EBP initiation. Unexpectedly, having an anxiety disorder was also associated with increased odds of EBP initiation.

Lower rates of EBP initiation associated with SMI may be due to stability of care factors (e.g., medication adherence) that may be necessary before engagement in first-line PTSD treatments. For those with a diagnosis of a SMI, there may also be other contraindications for EBP such as an increased risk of dissociation (29). EBP may be inappropriate for some individuals with SMI, and thus may explain why this population initiates EBP for PTSD less often; however, similar misperceptions about the inappropriateness of PTSD treatments for those with co-occurring SUD may also be occurring to those with co-occurring SMI. For example, results from a recent meta-analysis of EBP for individuals diagnosed with PTSD and co-occurring SMI (*N* = 300) were inconclusive regarding effectiveness for treating PTSD and psychotic symptoms. However, the authors reported that engaging in PTSD EBP was not inferior to waitlist control groups, and that in one study Eye Movement Desensitization and Reprocessing (EMDR) therapy demonstrated favorable preliminary evidence compared to a waitlist condition (30). More research is warranted for the use of EBP for Veterans with co-occurring SMI, SUD, and PTSD, particularly given that in the present sample over 20% of Veterans with PTSD-SUD also had a diagnosis of either bipolar disorder or a psychotic disorder. Research has also shown that adaptations to EBP have been effective for individuals with traumatic brain injuries (31, 32). We suggest that similar adaptations to EBP for those with co-occurring SMI could make these treatments more accessible for this population.

Notably, being 60 years and older was associated with a reduced likelihood of initiating EBP. This may be partially explained by generational factors, which may be associated with more stigmatizing views of mental health disorders, especially within military settings (33, 34). Substance use, and especially alcohol use is often seen as a normative way to cope with trauma within military culture (35, 36). Older Veterans may especially be reluctant to seek mental health treatment for these reasons. Additionally, older Veterans with both PTSD and SUD may also be managing more acute health issues and may not be able to engage in trauma-focused care. Recent increases in PTSD research and treatments, as well as recent VHA initiatives centered on Veteran reintegration after military service and exposure to mental health service availability may buffer stigma related to seeking mental health services in younger generations of Veterans (37). Continued efforts to reduce mental health stigma and improve targeted treatment engagement strategies for Veterans of various service eras may improve EBP initiation and general mental health service utilization. Integrated treatment approaches, including Primary Care Mental Health Integration, may improve access to PTSD care for populations with acute health care needs such as older adults (38).

Having a service connected disability was associated with a reduced likelihood of EBP initiation. PTSD is the most common mental health service connection disability (39). This finding is surprising given that PTSD treatment services are of little-to-no cost when Veterans receive service connected disability benefits, however; there are a few reasons that reduced EBP engagement may be seen in this subgroup. First, Veterans may fear losing their service connection disability status if they receive treatment for PTSD. Service connection provides Veterans compensation for living expenses and health benefits, and some Veterans may not be able to support themselves if their disability status changes. PTSD symptoms can significantly impact vocational functioning, and Veterans may be worried that if their symptoms return and they no longer have service connection benefits, they could be left without access to adequate resources and support. Relatedly, many service connected conditions are associated with other conditions such as chronic pain, and Veterans may fear these conditions will be exacerbated due to increasing distress related to exposure-based trauma therapies. Psychoeducation related to the overall positive outcomes associated with EBP for PTSD, and/or the effectiveness of concurrent treatment of pain while undergoing PTSD treatment may quell some of these concerns. Another possible reason for reduced rates of EBP initiation among service connected Veterans with PTSD-SUD may be limitations in outreach among newly service connected Veterans. One study assessed trajectories of EBP initiation before and after receiving a service connected disability for PTSD among a national sample of Veterans and found that treatment initiation was low both before and after receiving a PTSD-related service connection rating (40). This finding suggests that Veterans may wait significantly longer following service connection before initiating an EBP. More research is needed to understand why service connection status is associated with lower rates of EBP utilization. While addressing barriers to accessing treatment is complex and beyond the scope of this study, perhaps investment in programming that bolsters engagement in VHA care among Veterans going through Compensation and Pension examinations, or who are seeking support from Veteran Service Organizations for service connection applications, may partially improve treatment initiation.

Results showed lower initiation of EBP among female Veterans compared to males. Female Veterans have the highest rates of PTSD compared to civilians and male Veterans (41), and are more likely to experience MST compared to males, even controlling for underreporting (42, 43). MST can lead to experiences of institutional betrayal by systems representing the military, and hesitation to use VA services (44, 45). Additionally, survey research has indicated that female Veterans experience discrimination across VA settings, including sexual harassment in waiting rooms and microaggressions during appointments by healthcare providers (46–48), which may further reduce likelihood of initiating treatment. Moreover, societal expectations related to traditional gender roles have placed additional stigma on women diagnosed with SUD, as it may be viewed as neglecting family responsibilities, whereas substance use, and especially alcohol use is viewed as a more acceptable coping strategy for men (49). The stigma faced by female Veterans with SUD may also reduce treatment initiation within this subgroup. Further, factors such as childcare, caregiving for other adults, and transportation costs are more likely to be experienced by women, may be an additional burden preventing enrollment in EBP for PTSD (48). Regardless of the specific reasons, it is imperative that efforts are enacted to increase safety among female Veterans who utilize VHA services. These efforts should include development and implementation of policies, training and reporting systems, clearly defining harassment, and changing harassment norms to zero-tolerance within VHA (47, 50). Moreover, validating and addressing experiences of institutional betrayal among female Veterans may increase willingness to utilize VA health services (45).

Interestingly, a positive MST screen and being at high suicide risk were both associated with increased EBP initiation. The VHA requires that all Veterans who utilize VA care complete a MST annual screen and a suicide risk screen. Additionally, the VA offers resources such as a MST and Suicide Prevention Coordinator, which assist Veterans with positive MST and suicide risk screens to navigate services within VA specific to those experiences. PTSD is the most common mental health diagnosis to develop resulting from MST (51). MST is also associated with increased risk of substance use (52), and substance use increases suicide risk among those with PTSD (53–55). Given the risks associated with MST, suicide, and co-occurring PTSD-SUD, connecting Veterans to services such as EBP as early as possible is critical, and likely results in the increased rates of EBP initiation found in this study.

Finally, results also demonstrated that greater odds of EBP initiation were associated with anxiety disorder diagnoses. This finding may be associated with the overlap between PTSD and anxiety symptoms, and/or overlap between substance-related withdrawal and craving and anxiety disorders. The categorical nature of DSM-5 diagnostics can lend itself to overlapping symptoms across diagnoses and multiple simultaneous diagnoses. For this reason, it is difficult to conclude from the EHR data whether having an additional anxiety disorder is secondary to PTSD or SUD symptoms, or the degree to which PTSD, SUD, and anxiety symptoms overlap. Perhaps having an anxiety disorder can more easily alert clinicians to the possibility of a subsequent PTSD diagnosis, and therefore these individuals may be more likely to receive services. Further, distress associated with anxiety disorders may motivate individuals to seek treatment.

These data highlight differences and similarities in factors associated with initiation of EBP for PTSD between those with and without co-occurring SUD, as well as the ways health services factors differ for Veterans with PTSD-SUD. Given the increased risk of housing instability, justice involvement, greater psychiatric and physical impairment, and cultural stigma among those with PTSD-SUD (2–4, 22), consideration of how these additional barriers may impact EBP initiation and adjusting efforts to better engage subpopulations of Veterans with PTSD-SUD is warranted. For example, modifications to CPT have demonstrated reductions in PTSD and secondary outcomes comparable to standard CPT (56, 57), indicating that manualized EBP can be flexible to better meet the needs of subpopulations who are less likely to initiate treatment (58–60). Moreover, bolstering screening procedures and engagement with treatment coordinators to better identify Veterans with a lower likelihood of EBP initiation and connect them to treatment may also enhance EBP initiation among those with co-occurring SUD.

## Limitations

While research suggests that EMDR and COPE are potentially beneficial treatments for PTSD and PTSD-SUD, VHA does not currently have EHR templates for these interventions. Therefore, we were unable to identify and analyze treatment data related to these specific EBPs from the VHA EHR using the EHR template methods we employed in this study. Using EHR data poses an additional set of limitations. For example, it is difficult to examine certain variables like nuances of Veteran-identified gender, race, and ethnicity, partnerships outside of marriage, and other aspects of social support. For this reason, more research is needed on intersecting identities, how these may impact health service utilization, and why. Further, because we were unable to randomize and control for many potentially confounding variables using EHR data, making comparisons between EBPs impossible. Thus, these results should be interpreted as descriptive, and not as causal. Finally, initiation in EBP does not imply that an individual has completed a full course of EBP; thus, the implications of this study are solely limited to initiation. Future research should investigate which factors predict sustained engagement in and completion of EBP after initiation to optimize outcomes among those with PTSD-SUD.

## Conclusion

Identifying EBP initiation patterns, patient characteristics, and health service factors that facilitate or reduce the likelihood of EBP initiation among those with PTSD-SUD may provide insight into more efficient and effective treatment pathways. This largescale, national, EHR-based research suggests that some patterns of initiation among Veterans with PTSD-SUD are similar to those with PTSD alone. Notably, rates of initiation were similar across these groups, suggesting that the VA is providing relatively equal opportunities for Veterans with PTSD to initiate EBP regardless of the presence of co-occurring SUD.

## Data Availability

The raw data supporting the conclusions of this article will be made available by the authors, without undue reservation.
